# Longitudinal assessment of systemic steroid therapy on hyperinflammatory endothelial biomarker profiles and serology responses of COVID-19 patients

**DOI:** 10.1186/s12967-022-03583-5

**Published:** 2022-09-08

**Authors:** Jonathan T. Sims, Ching-Yun Chang, Josh Poorbaugh, Montanea Daniels, Stephanie L. Beasley, Lin Zhang, George H. Rodgers, Fabio Lena, Leonardo G. Lacerenza, Bruno Sposato, Annabelle Dupont, Sophie Susen, Giacomo Casalini, Mario Corbellino, Justin Stebbing, Venkatesh Krishnan

**Affiliations:** 1 Eli Lilly and Company, Lilly Corporate Center, Indianapolis, IN 46285 USA; 2 Department of Pharmaceutical Medicine, Misericordia Hospital, Grosseto, Italy; 3 Inserm, CHU Lille, Institut Pasteur de Lille, U1011-EGID, University of Lille, 59000 Lille, France; 4 Luigi Sacco Department of Clinical and Biomedical Sciences, University of Milan, Milan, Italy; 5 Division of Infectious Diseases, ASST Fatebenefratelli Sacco, Milan, Italy; 6 Department of Surgery and Cancer, Imperial College, London, UK

**Keywords:** COVID-19, CXCL9, Cytokine, Dexamethasone, Inflammation, Serology

This first of its kind study provides objective context to the potential mechanism of action of corticosteroid use by connecting inflammatory biomarkers to IgG levels for the SARS-CoV-2 spike protein antigens and neutralization of ACE2 binding within patients across 3 institutions from Italy and France who received corticosteroids (dexamethasone, n = 5, or prednisolone, n = 1) or usual standard of care (SOC, n = 22) therapy. The median Ordinal Scale (WHO) upon admission was OS-5 and no difference in days from symptom onset or Ordinal scale at study entry was observed between these 2 groups (Additional file 1: Table S1, Fig. S1). Utilizing Olink multiplex technology and IL-19 ELISA, we assessed 185 analytes in the circulation of COVID-19 patients along with Luminex-based measurement of 10 immunoglobulins, including neutralization assessment (Additional file 2).

We observed prominent dysregulation of IL-8, CCL7/MCP-3, S100A12/ENRAGE, and IL-6 in the circulation of these patient cohorts relative to 12 age/sex-matched healthy controls (HC) (Fig. 1A). IL-6 correlated modestly with baseline Ordinal scale and the broader inflammatory biomarker profile was characterized by strong correlations to circulating neutrophils and serum creatinine (Fig. 1A). Levels of TNFRSF10A, IL-10RA, CXCL9, TRAILR2, IL-18, and TNFa negatively correlated with SpO2 percentage and positively correlated with Ordinal Scale at admission emphasizing the potentially important role of these molecular pathways within hospitalized patients (Fig. 1A) [1].

**Fig. 1:**
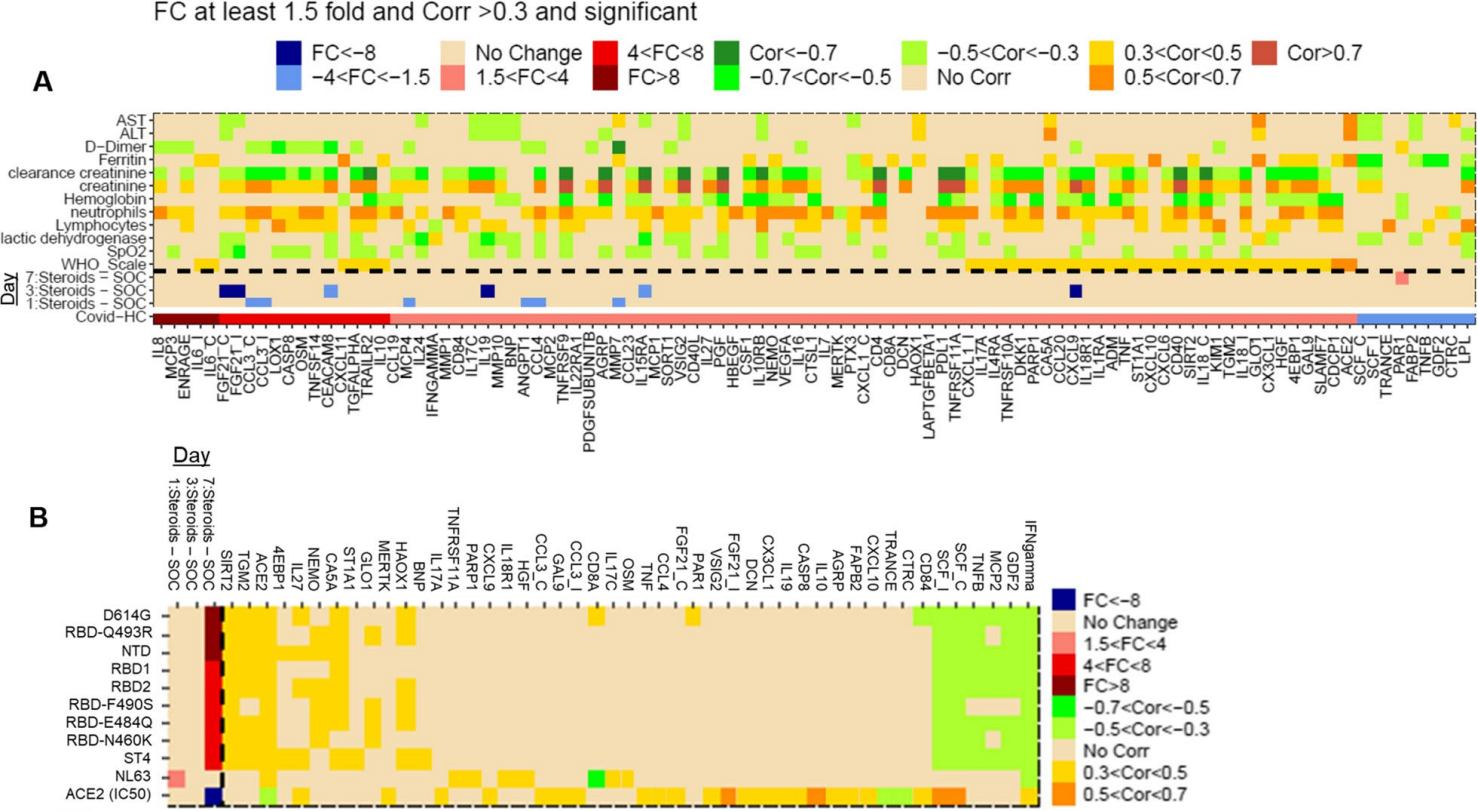
**A** (lower) Heatmap analysis comparing fold change for protein biomarker levels in baseline samples of COVID-19 patients versus age/sex-matched healthy controls (HC). Analytes are ordered along the x-axis by P-value significance (P < 0.05) based on their positive and negative FC (> 1.5×) relative to HC. **A** (middle) Fold change of analytes dysregulated in baseline COVID-19 patients relative to HCs over time between Steroid and SOC treated patients. **A** (top) Correlations of biomarkers and clinical assessments (i.e., WHO Ordinal scale, inflammatory signals (d-dimer, Ferritin), and blood cell counts) are shown for baseline samples of COVID-19 patients. **B** (left) Heatmap analysis of fold change of IgG levels for the SARS-CoV-2 spike protein antigens over time between Steroid and SOC treated patients. **B** (right) Correlations of biomarkers with IgG levels for SARS-CoV-2 spike protein antigens over time. Red or blue represents significantly upregulated or downregulated markers, respectively, between 1.5× and 4× (light shade), 4×–8× (medium shade), or > 8× (dark shade). Orange or green represents significantly positive or negative correlated markers, respectively, between 0.3 < Cor < 0.5 (light shade), 0.5 < Cor < 0.7 (medium shade), or Cor > 0.7 (dark shade). “_I” or “_C” refers to analytes measured by Olink Inflammation I panel or Cardiovascular II panel, respectively

Focusing on markers differentially regulated in COVID-19 patients, we observed a unique biomarker profile in patients depending on their therapeutic journey. Analytes elevated in baseline COVID-19, FGF21, CCL3, CCL4, CEACAM8, CCL13/MCP4, IL-19, IL15RA, ANGPT1, MMP7, and CXCL9, all decreased more in corticosteroid-treated patients relative to SOC (Fig. 1A).

We observed serology titers for all SARS-CoV-2 antigens with RBD mutant proteins increasing or remaining relatively unchanged within steroid-treated patients relative to the SOC-treated patients over the course of 7 days in this small study, mirrored by an increase in neutralization of the RBD-ACE2 interaction in the same steroid-treated patients (Fig. 1B). Further research into the mechanism of action of corticosteroid-reduced IgG levels will need to occur, but in this study, there was no detrimental impact on antibody responses to COVID-19 infection. Within these patients, repeated measured correlations demonstrated inverse correlation of ACE2 neutralization IC50 not only with ACE2, but also TNFSF11/TRANCE protein in the serum (Fig. 1B). This data argues that circulating antibodies with more potent neutralization (lower IC50) are found in patients with higher serum levels of ACE2 and TNFSF11/TRANCE, the former known to be shed by interactions with viral spike protein and the latter to decrease when viral pathogenesis is most robust [2, 3]. Related to the broad suppressive nature of corticosteroids, certain analytes reduced in circulation of COVID-19 patients, such as SCF and GDF2, were inversely correlated with serological IgG response markers (Fig. 1B). Intriguingly, a unique inflammatory biomarker profile was observed to correlate with the seasonal coronavirus protein control (NL63) relative to the profile linked to SARS-CoV-2 antigens and related RBD mutant proteins (Fig. 1B). Positive correlations were observed for IL-27, MERTK, IL-17A, CXCL9, CCL3, GAL9, TNF, CCL4, FGF21, VSIG2, DCN, CX3CL1, IL-19, CASP8, IL-10, AGRP, FABP2, CXCL10, CD84, SCF, and IFNg with ACE2 neutralization IC50 values (Fig. 1B). Higher ACE2 neutralization IC50 values indicate less potency of the antibody response and hence the observed increase in many proinflammatory signals (e.g. IL-19, TNF, CXCL9, and IFNg). This tight regulatory phenomenon between inflammatory biomarkers and serology was clearly evident within the tested timeframe of 7 days post corticosteroid treatment.

The correlation of COVID-19 inflammatory markers, which we previously linked to severe disease (e.g., IFNg, TNF, CXCL9, and IL-10), with higher IC50 neutralization values may demonstrate poor levels of ACE2/RBD interference in patients experiencing the hyperinflammatory endothelial-linked cytokine storm of COVID-19 [4].

Importantly, the elevation of markers indicative of innate immune activation (CCL13/MCP4, CCL3, CCL4, CXCL9) are reduced in steroid-treated patients. We observed that CXCL9 is an important COVID-19 biomarker describing immunological responses in corticosteroid-treated patients based on its correlation to Ordinal scale at baseline, decreased levels over time in corticosteroid-treated patients, and correlation with ACE2 neutralization IC50 values; therefore, warranting further evaluation and clinical monitoring within patients treated with corticosteroids. However, key systemic inflammatory cytokines linked to adaptive immune responses elevated in COVID-19 vs. HC are not differentially regulated in steroid vs SOC-treated patients, demonstrating that early effective targeted therapies against these will be most successful in rapidly reducing the inflammatory burden that severe patients experience.

## Supplementary Information

**Additional file 1: Table S1.** Baseline characteristics of COVID-19 patients. **Table S2.** Recombinant proteins used in serology measurements. **Figure S1**. **A** Patient classification based on therapeutic decision prior to collection of first sample as either steroids or SOC (non-steroid). **B**, **C** Graphical box plot representations of the time (days) between a patient’s symptom onset and collection of first sample (B) or Ordinal Scale classification at admission (C) between steroid and SOC patient groups.

**Additional file 2.** Additional Materials and Methods.

## Data Availability

The data from the current study are available from the corresponding author on reasonable request.
