# Supplementary material for: Longitudinal assessment of systemic steroid therapy on hyperinflammatory endothelial biomarker profiles and serology responses of COVID-19 patients
Source: J Transl Med. 2022 Sep 8;20:411. doi: 10.1186/s12967-022-03583-5 (PMC9458306; doi:10.1186/s12967-022-03583-5)
Supplement: Supplementary file 1 — Additional file 1: Table S1. Baseline characteristics of COVID-19 patients. Table S2. Recombinant proteins used in serology measurements. Figure S1. A Patient classification based on therapeutic decision prior to collection of first sample as either steroids or SOC (non-steroid). B, C Graphical box plot representations of the time (days) between a patient’s symptom onset and collection of first sample (B) or Ordinal Scale classification at admission (C) between steroid and SOC patient groups. [file 12967_2022_3583_MOESM1_ESM.pptx]

## Slide 1
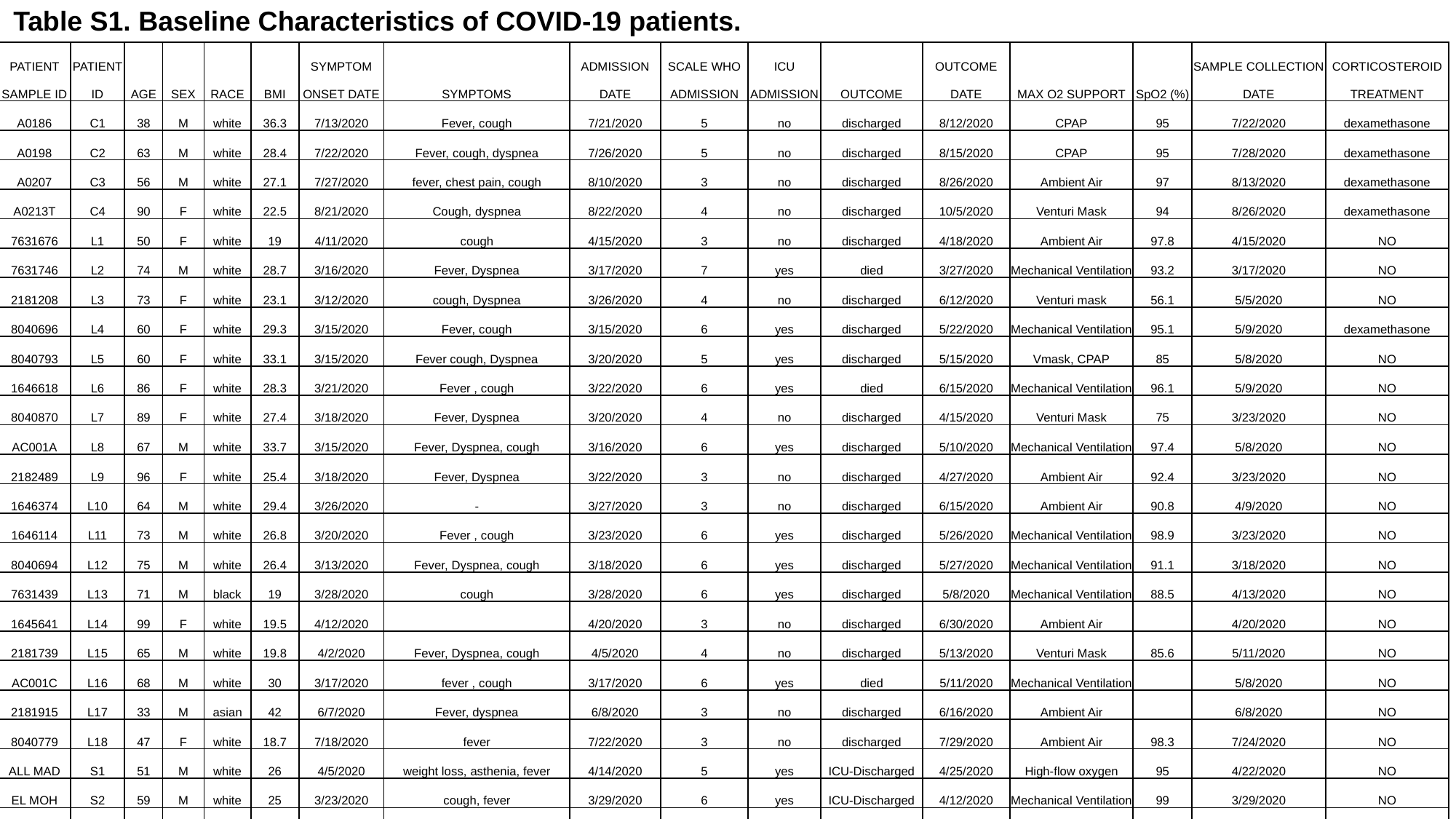

Table S1. Baseline Characteristics of COVID-19 patients.
| PATIENT SAMPLE ID | PATIENT ID | AGE | SEX | RACE | BMI | SYMPTOM ONSET DATE | SYMPTOMS | ADMISSION DATE | SCALE WHO ADMISSION | ICU ADMISSION | OUTCOME | OUTCOME DATE | MAX O2 SUPPORT | SpO2 (%) | SAMPLE COLLECTION DATE | CORTICOSTEROID TREATMENT |
| --- | --- | --- | --- | --- | --- | --- | --- | --- | --- | --- | --- | --- | --- | --- | --- | --- |
| A0186 | C1 | 38 | M | white | 36.3 | 7/13/2020 | Fever, cough | 7/21/2020 | 5 | no | discharged | 8/12/2020 | CPAP | 95 | 7/22/2020 | dexamethasone |
| A0198 | C2 | 63 | M | white | 28.4 | 7/22/2020 | Fever, cough, dyspnea | 7/26/2020 | 5 | no | discharged | 8/15/2020 | CPAP | 95 | 7/28/2020 | dexamethasone |
| A0207 | C3 | 56 | M | white | 27.1 | 7/27/2020 | fever, chest pain, cough | 8/10/2020 | 3 | no | discharged | 8/26/2020 | Ambient Air | 97 | 8/13/2020 | dexamethasone |
| A0213T | C4 | 90 | F | white | 22.5 | 8/21/2020 | Cough, dyspnea | 8/22/2020 | 4 | no | discharged | 10/5/2020 | Venturi Mask | 94 | 8/26/2020 | dexamethasone |
| 7631676 | L1 | 50 | F | white | 19 | 4/11/2020 | cough | 4/15/2020 | 3 | no | discharged | 4/18/2020 | Ambient Air | 97.8 | 4/15/2020 | NO |
| 7631746 | L2 | 74 | M | white | 28.7 | 3/16/2020 | Fever, Dyspnea | 3/17/2020 | 7 | yes | died | 3/27/2020 | Mechanical Ventilation | 93.2 | 3/17/2020 | NO |
| 2181208 | L3 | 73 | F | white | 23.1 | 3/12/2020 | cough, Dyspnea | 3/26/2020 | 4 | no | discharged | 6/12/2020 | Venturi mask | 56.1 | 5/5/2020 | NO |
| 8040696 | L4 | 60 | F | white | 29.3 | 3/15/2020 | Fever, cough | 3/15/2020 | 6 | yes | discharged | 5/22/2020 | Mechanical Ventilation | 95.1 | 5/9/2020 | dexamethasone |
| 8040793 | L5 | 60 | F | white | 33.1 | 3/15/2020 | Fever cough, Dyspnea | 3/20/2020 | 5 | yes | discharged | 5/15/2020 | Vmask, CPAP | 85 | 5/8/2020 | NO |
| 1646618 | L6 | 86 | F | white | 28.3 | 3/21/2020 | Fever , cough | 3/22/2020 | 6 | yes | died | 6/15/2020 | Mechanical Ventilation | 96.1 | 5/9/2020 | NO |
| 8040870 | L7 | 89 | F | white | 27.4 | 3/18/2020 | Fever, Dyspnea | 3/20/2020 | 4 | no | discharged | 4/15/2020 | Venturi Mask | 75 | 3/23/2020 | NO |
| AC001A | L8 | 67 | M | white | 33.7 | 3/15/2020 | Fever, Dyspnea, cough | 3/16/2020 | 6 | yes | discharged | 5/10/2020 | Mechanical Ventilation | 97.4 | 5/8/2020 | NO |
| 2182489 | L9 | 96 | F | white | 25.4 | 3/18/2020 | Fever, Dyspnea | 3/22/2020 | 3 | no | discharged | 4/27/2020 | Ambient Air | 92.4 | 3/23/2020 | NO |
| 1646374 | L10 | 64 | M | white | 29.4 | 3/26/2020 | - | 3/27/2020 | 3 | no | discharged | 6/15/2020 | Ambient Air | 90.8 | 4/9/2020 | NO |
| 1646114 | L11 | 73 | M | white | 26.8 | 3/20/2020 | Fever , cough | 3/23/2020 | 6 | yes | discharged | 5/26/2020 | Mechanical Ventilation | 98.9 | 3/23/2020 | NO |
| 8040694 | L12 | 75 | M | white | 26.4 | 3/13/2020 | Fever, Dyspnea, cough | 3/18/2020 | 6 | yes | discharged | 5/27/2020 | Mechanical Ventilation | 91.1 | 3/18/2020 | NO |
| 7631439 | L13 | 71 | M | black | 19 | 3/28/2020 | cough | 3/28/2020 | 6 | yes | discharged | 5/8/2020 | Mechanical Ventilation | 88.5 | 4/13/2020 | NO |
| 1645641 | L14 | 99 | F | white | 19.5 | 4/12/2020 | | 4/20/2020 | 3 | no | discharged | 6/30/2020 | Ambient Air | | 4/20/2020 | NO |
| 2181739 | L15 | 65 | M | white | 19.8 | 4/2/2020 | Fever, Dyspnea, cough | 4/5/2020 | 4 | no | discharged | 5/13/2020 | Venturi Mask | 85.6 | 5/11/2020 | NO |
| AC001C | L16 | 68 | M | white | 30 | 3/17/2020 | fever , cough | 3/17/2020 | 6 | yes | died | 5/11/2020 | Mechanical Ventilation | | 5/8/2020 | NO |
| 2181915 | L17 | 33 | M | asian | 42 | 6/7/2020 | Fever, dyspnea | 6/8/2020 | 3 | no | discharged | 6/16/2020 | Ambient Air | | 6/8/2020 | NO |
| 8040779 | L18 | 47 | F | white | 18.7 | 7/18/2020 | fever | 7/22/2020 | 3 | no | discharged | 7/29/2020 | Ambient Air | 98.3 | 7/24/2020 | NO |
| ALL MAD | S1 | 51 | M | white | 26 | 4/5/2020 | weight loss, asthenia, fever | 4/14/2020 | 5 | yes | ICU-Discharged | 4/25/2020 | High-flow oxygen | 95 | 4/22/2020 | NO |
| EL MOH | S2 | 59 | M | white | 25 | 3/23/2020 | cough, fever | 3/29/2020 | 6 | yes | ICU-Discharged | 4/12/2020 | Mechanical Ventilation | 99 | 3/29/2020 | NO |
| MOU JEA | S3 | 75 | M | white | 24 | 3/30/2020 | fever | 4/9/2020 | 4 | yes | ICU-Discharged | 4/16/2020 | Oxygen by mask | 96 | 4/15/2020 | NO |
| PAU ALA | S4 | 73 | M | white | 25 | 4/2/2020 | flu syndrome, dyspnea | 4/6/2020 | 5 | yes | ICU-Discharged | 4/14/2020 | High-flow oxygen | 95 | 4/13/2020 | NO |
| REP FRA | S5 | 71 | M | white | 25 | 4/8/2020 | fever, dyspnea, anosmia | 4/16/2020 | 4 | yes | ICU-Discharged | 4/20/2020 | Oxygen by mask | 98 | 4/16/2020 | NO |
| LER EMM | S6 | 56 | M | white | 37 | 3/15/2020 | fever, dyspnea, diarrhea, cough | 3/25/2020 | 7 | yes | ICU-Discharged | 6/8/2020 | Mechanical Ventilation | 99 | 4/4/2020 | methylprednisolone |

## Slide 2
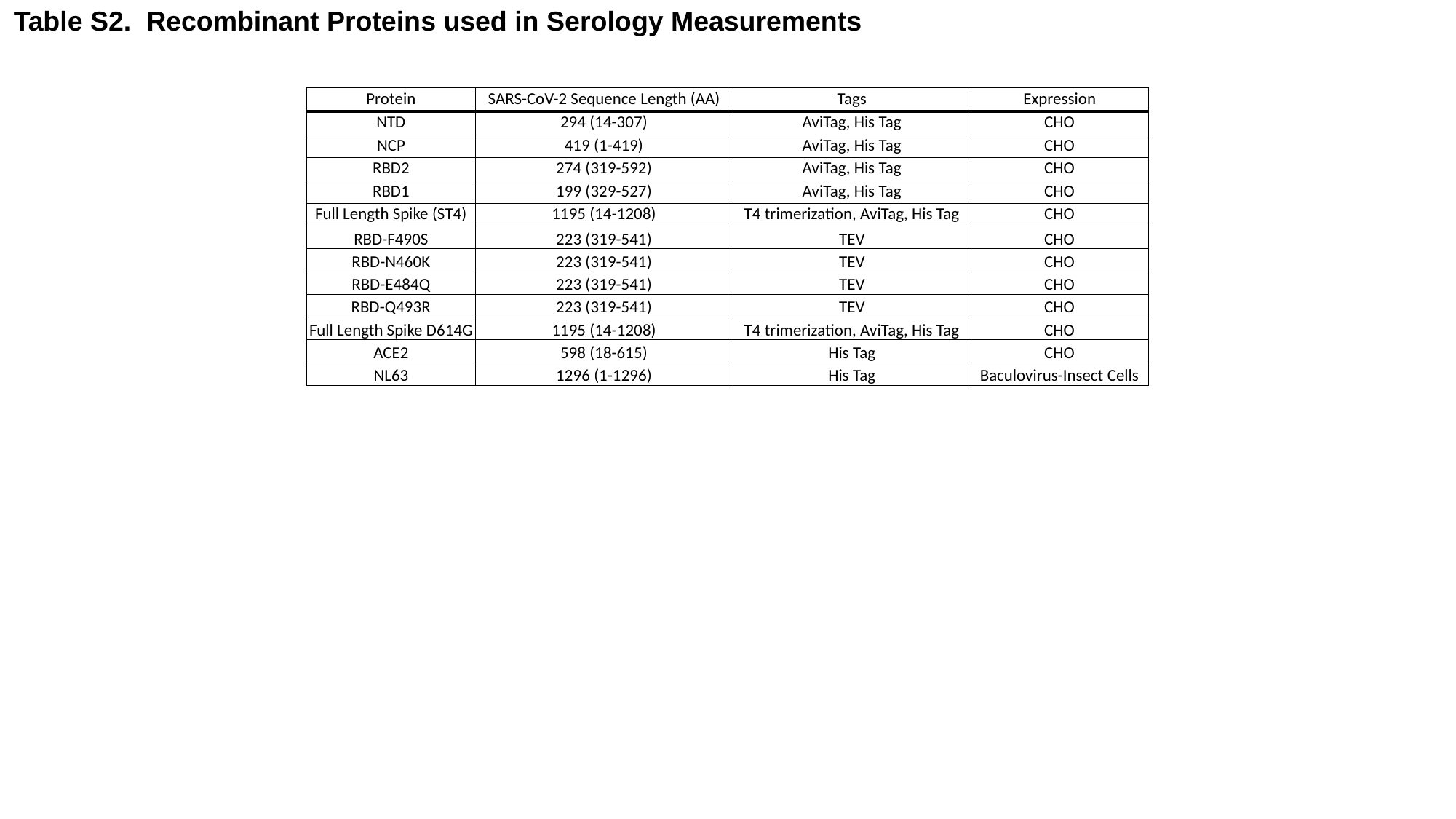

Table S2. Recombinant Proteins used in Serology Measurements
| Protein | SARS-CoV-2 Sequence Length (AA) | Tags | Expression |
| --- | --- | --- | --- |
| NTD | 294 (14-307) | AviTag, His Tag | CHO |
| NCP | 419 (1-419) | AviTag, His Tag | CHO |
| RBD2 | 274 (319-592) | AviTag, His Tag | CHO |
| RBD1 | 199 (329-527) | AviTag, His Tag | CHO |
| Full Length Spike (ST4) | 1195 (14-1208) | T4 trimerization, AviTag, His Tag | CHO |
| RBD-F490S | 223 (319-541) | TEV | CHO |
| RBD-N460K | 223 (319-541) | TEV | CHO |
| RBD-E484Q | 223 (319-541) | TEV | CHO |
| RBD-Q493R | 223 (319-541) | TEV | CHO |
| Full Length Spike D614G | 1195 (14-1208) | T4 trimerization, AviTag, His Tag | CHO |
| ACE2 | 598 (18-615) | His Tag | CHO |
| NL63 | 1296 (1-1296) | His Tag | Baculovirus-Insect Cells |

## Slide 3
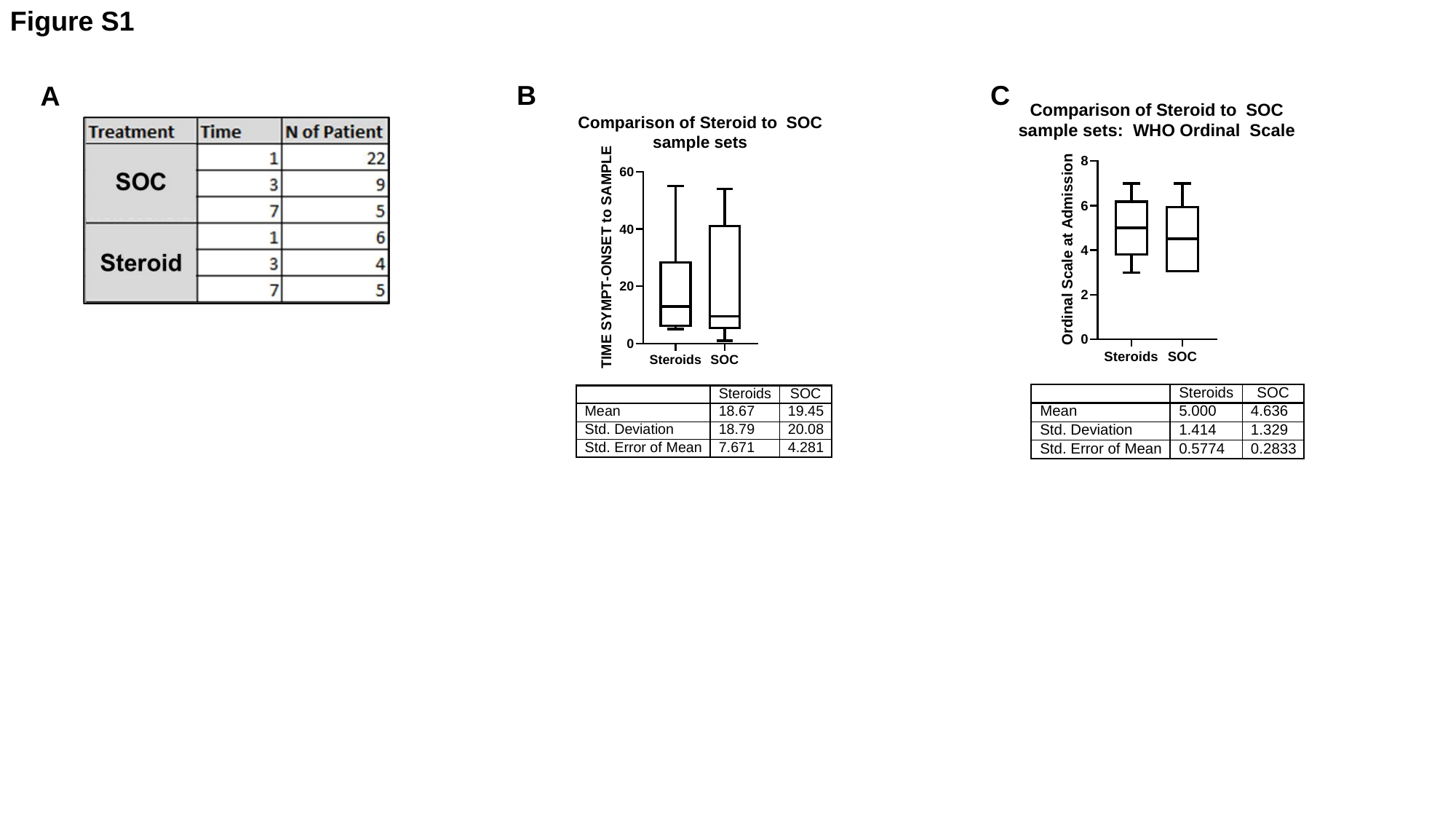

Figure S1
B
C
A
