# Supplementary material for: Longitudinal assessment of systemic steroid therapy on hyperinflammatory endothelial biomarker profiles and serology responses of COVID-19 patients
Source: J Transl Med. 2022 Sep 8;20:411. doi: 10.1186/s12967-022-03583-5 (PMC9458306; doi:10.1186/s12967-022-03583-5)
Supplement: Supplementary file 2 — Additional file 2. Additional Materials and Methods. [file 12967_2022_3583_MOESM2_ESM.docx]

**ADDITIONAL MATERIALS**

**METHODS**

**Patients and Samples**

This study included a cohort of 28 patients requiring supplemental oxygen. 6 received corticosteroids (Steroids) prior to first sample collection, while 22 received a variety of standard of care (SOC) therapeutic regimen ranging from antibiotics to antivirals (Figure S1A). The median Ordinal Scale (WHO) upon admission was OS5. No difference was observed in Ordinal scale at admission or mean time to symptom onset between Steroids or SOC groups (Figure S1B, C). 12 age/sex-matched healthy controls were included for comparison.

**ELISA and multiplex immunoassays**

*Protein Biomarker Assessments*
Serum samples were analyzed with the Olink Inflammation I and Cardiovascular II Proseek multiplex assays (Uppsala, Sweden), a proximity extension assay (PEA) technology that utilizes oligonucleotide-labeled antibody probes, according to manufacturer’s specifications. The levels of 184 analyte-specific deoxyribonucleic acid (DNA) amplicons were quantified for each patient on the Fluidigm Biomark HD (San Francisco, CA). IL-19 was assessed as previously described [4].

*Luminex Serology and Neutralization methodology*

Fluorescently-dyed MagPlex-Microspheres (Luminex xMAP) were used to separate multiple conjugated protein antigens into spectrally distinct regions allowing for the simultaneous detection and quantitation of antibodies against these proteins. Additionally, endogenous neutralization of the spike protein in patient sample was measured by detecting the ability of a PE-labeled RBD to bind to a recombinant ACE2-conjugated bead.

For serological/neutralization testing, serum samples were titered in phosphate buffered saline-high salt solution (PBS-HS; 0.01 M PBS, 1% BSA, 0.02% Tween, 300 mM NaCl) and mixed with antigen-conjugated microspheres (4 SARS-CoV-2 antigens (NTD, RBD1, RBD2, and full length spike (ST4)), 5 mutant proteins (RBD-F490S, RBD-N460K, RBD-E484Q, RBD-Q493R, and full length spike D614G) and a seasonal coronavirus family member (NL63), as well as phycoerythrin (PE)-conjugated RBD antigen (50ng/mL)) for 45 minutes (Table S2). Subsequently, beads were separated from supernatant using a magnet with 75uL transferred to a new plate containing ACE2-conjugated beads. The remaining beads were washed and combined with secondary PE-conjugated antibody (αIgG, #109-115-098, Jackson Labs). Both plates were incubated for 45 minutes at RT. Finally, plates were washed (3x) with PBS–low salt solution (PBS-LS; 0.01 M PBS, 1% BSA, 0.02% Tween) and read using a Luminex FlexMap3D System.

**Statistical Analyses**

Titer is commonly defined as the smallest dilution above the cut point or the dilution factor at the cut point based on an interpolation of assay values that straddle the cut point. In the serology assay, we used the latter method to calculate the titers. To calculate potency of the neutralization component of the Luminex assay, a 4-parameter logistic regression was used to calculate the absolute IC50. Samples with negative maximum percentage inhibition were excluded from further analysis, while IC50s of samples with maximum percentage inhibition less than 50% were imputed to a large number.

Spearman correlation coefficient was calculated between the titers, IC50s, protein biomarkers, and clinical measures accounting for multiplicity adjustment and false discovery rate as previously described [4]. A mixed-effect model was used to assess time effect of treatment accounting for age, sex, and multiplicity adjustments as previously described [4].
